# Comprehensive analysis of fatty acid and lactate metabolism–related genes for prognosis value, immune infiltration, and therapy in osteosarcoma patients

**DOI:** 10.3389/fonc.2022.934080

**Published:** 2022-09-02

**Authors:** Zhouwei Wu, Tao Han, Haohan Su, Jiangwei Xuan, Xinwei Wang

**Affiliations:** ^1^ Department of Orthopaedic Surgery, the Second Affiliated Hospital of Wenzhou Medical University, Wenzhou, China; ^2^ Department of Orthopaedic Surgery, Zhuji Affiliated Hospital of Wenzhou Medical University, Zhuji, China

**Keywords:** fatty acid metabolism, lactate metabolism, metastasis, osteosarcoma, prognosis, immunity

## Abstract

Osteosarcoma is the most frequent bone tumor. Notwithstanding that significant medical progress has been achieved in recent years, the 5-year overall survival of osteosarcoma patients is inferior. Regulation of fatty acids and lactate plays an essential role in cancer metabolism. Therefore, our study aimed to comprehensively assess the fatty acid and lactate metabolism pattern and construct a fatty acid and lactate metabolism–related risk score system to predict prognosis in osteosarcoma patients. Clinical data and RNA expression data were downloaded from the Therapeutically Applicable Research to Generate Effective Treatments (TARGET) and Gene Expression Omnibus (GEO) databases. We used the least absolute shrinkage and selection operator (LASSO) and Cox regression analyses to construct a prognostic risk score model. Relationships between the risk score model and age, gender, tumor microenvironment characteristics, and drug sensitivity were also explored by correlation analysis. We determined the expression levels of prognostic genes in osteosarcoma cells *via* Western blotting. We developed an unknown fatty acid and lactate metabolism–related risk score system based on three fatty acid and lactate metabolism–related genes (SLC7A7, MYC, and ACSS2). Survival analysis showed that osteosarcoma patients in the low-risk group were likely to have a better survival time than those in the high-risk group. The area under the curve (AUC) value shows that our risk score model performs well in predicting prognosis. Elevated fatty acids and lactate risk scores weaken immune function and the environment of the body, which causes osteosarcoma patients’ poor survival outcomes. In general, the constructed fatty acid and lactate metabolism–related risk score model can offer essential insights into subsequent mechanisms in available research. In addition, our study may provide rational treatment strategies for clinicians based on immune correlation analysis and drug sensitivity in the future.

## Introduction

Osteosarcoma is the most frequent solid malignancy of bone, and it is common in children and adolescents (1). The incidence of osteosarcoma is approximately 2–3 million per year in the general population and about 8–11 million annually in the 15–19 years of age population (2). Metastasis is also common in osteosarcoma patients, approximately 15–30% of osteosarcoma patients with metastasis (3, 4). Osteosarcoma often accompanied the progression of metastatic disease if it was untreated (2). Before the introduction of polychemotherapy, more than 90% of patients with osteosarcoma died from lung metastases. Although the general management of osteosarcoma with metastasis has improved dramatically, the prognosis of patients with metastatic osteosarcoma is still inferior (5). The long-term survival is nearly less than 20% among patients with metastatic osteosarcoma (2). Therefore, developing accurate and reliable biological indicators for prognostic prediction and individualized treatment is urgent.

Due to the proliferation of cancer cells, the overall cancer microenvironment is characterized by high oxidation, acidity, malnutrition, and hypoxia. Therefore, tumor cells have different metabolic characteristics than normal cells, which leads to a significant difference in metabolites in cancer cells with normal cells (6, 7). Recently, fatty acid metabolism, essential for many biological activities, has attracted much attention in cancer cells (8, 9). Fatty acid metabolism is closely related to cancer development. Similarly, lactate, a significant carbon source for cell metabolism, has been pointed out that plays an essential role in cancer development, maintenance, and therapeutic response (10, 11). Lactate metabolism is also related to cancer metabolism and prognosis. Lactate has been proved to regulate all aspects of cancer cell behavior (12). However, the association between fatty acid metabolism and lactate metabolism–related genes and the prediction of osteosarcoma has not been illustrated.

As we all know, the prognostic prediction model of cancers has a critical role in clinical applications and improves patients’ prognostic management. Fatty acid and lactate metabolism plays an essential role in the progress and development of cancer. Therefore, we were trying to comprehensively assess the fatty acid metabolism and lactate metabolism pattern and construct a fatty acid and lactate prognostic risk score system to predict prognosis in osteosarcoma patients. The prognostic risk score model system could predict the survival of osteosarcoma. Moreover, we also investigated the relationship between the prognostic risk score system and tumor microenvironments (TMEs) cell-infiltrating characteristics. Our study may provide a novel perspective for exploring osteosarcoma’s metabolic mechanism and treatment.

## Materials and methods

### Data collection

We extracted RNA sequencing (RNA-seq) and corresponding clinical data of 84 osteosarcoma patients (63 cases without metastasis and 21 cases with metastasis) from the Therapeutically Applicable Research to Generate Effective Treatments (TARGET) database. We downloaded the RNA-seq data and clinical information of 53 osteosarcoma patients of the external validation cohort from the Gene Expression Omnibus (GEO) database (GSE21257) as an external validation cohort. We extracted 345 fatty acid metabolism– and lactate metabolism–related genes from the Molecular Signatures Database (MSigDB; https://www.gsea-msigdb.org/gsea/msigdb/index.jsp) and previous studies (shown in **Supplementary Table S1**) (13–15).

### Identification of differentially expressed fatty acid and lactate metabolism–related genes

Before comparing, we first normalized expression data to fragment per kilobase million (FPKM) values. We used the “limma” R package to find different expressions of fatty acid and lactate metabolism–related genes. Then we constructed a correlation network for different expression of fatty acid and lactate metabolism–related genes. To explore the connections between the expression of the 18 fatty acid and lactate metabolism–related differentially expressed genes (DEGs) and osteosarcoma, we performed a consensus clustering analysis with 84 osteosarcoma patients.

### Development and validation of the fatty acid and lactate metabolism–related genes prognostic model

Univariable Cox regression analysis was used to assess the relationship between each gene and prognosis in the TARGET cohort. It could provide the prognostic score for fatty acid and lactate metabolism–related genes. A *p* < 0.05 was set as criterion for genes included for further analysis. The regression of the least absolute shrinkage and selector operation (LASSO) was conducted to shrink the potential genes and build the prognostic prediction model. Then, non-zero regression coefficients were conducted in the TARGET cohort to variables for multivariable Cox regression analysis and further established the fatty acid metabolism and lactate metabolism risk score. The formula calculating for the risk score was provided in a previous study (16). High-risk and low-risk score groups were divided according to the median of the fatty acid metabolism and lactate metabolism risk score. Kaplan–Meier survival curve was conducted to show the prognosis of the two groups, followed by the log-rank test to show a significant difference. We calculated the area under the curve (AUC) value to evaluate the performance of the risk score system. Finally, the fatty acid and lactate metabolism–risk score system was externally validated in the GSE21257 validation cohort.

### Prognostic analysis of the risk score and development of a nomogram

We combined clinical information (age, gender, and metastatic status) of patients in the TARGET cohort with the risk score to include in univariate and multivariable Cox regression analysis. A nomogram was built to predict the prognosis of osteosarcoma patients based on the multivariable Cox regression analysis results. Calibration curves also evaluated the performance of the nomogram in the GSE21257 cohort.

### Functional enrichment analysis

To further explore the differences in the gene functions and pathways between the subgroups categorized by the risk model, we utilized the “limma” R package to extract the DEGs by applying the criteria FDR < 0.05 and |log_2_FC| ≥ 1. Gene ontology (GO) enrichment analysis and Kyoto Encyclopaedia of Genes and Genomes (KEGG) pathway analysis were performed based on these DEGs. The single-sample Gene Set Enrichment Analysis (ssGSEA) was conducted in high- and low-risk groups further to explore the infiltrating scores of immune cells and activity of immune-related pathways. At the same time, Benjamini–Hochberg (BH) correction method was used to calculate the adjusted *p*-value.

### Tumor immune microenvironment

We evaluated the cell infiltration levels in osteosarcoma *via* calculating the immune score and stromal score. The “estimate” R package can generate immune and stromal scores (17). We utilized the estimate algorithm to calculate the infiltration levels of immune and stromal cells. We applied Spearman correlation analysis to analyze the relationships between risk score and immune and stromal cells.

### Drug susceptibility analysis

We download the transcriptional expression of NCI-60 human cancer cell lines. We used Pearson correlation analysis to determine the correlation between predictive genes and drug sensitivity.

### Cell lines and cultures

A human osteoblast cell line (hFOB1.19) and two human osteosarcoma cell lines (U20S and 143B) were purchased from the National Collection of Authenticated Cell Cultures (Shanghai, China). The cells were cultured in Dulbecco’s modified Eagle’s medium (DMEM, Gibco, Thermo Fisher Scientific, MA, the USA) containing 1% penicillin/streptomycin (Thermo Fisher Scientific, MA, the USA) and 10% fetal bovine serum (FBS, Gibco, Thermo Fisher Scientific, MA, the USA). We cultured the human osteoblast cell line at 34°C with 5% CO_2_ and the osteosarcoma cell lines at 37°C with 5% CO_2_.

### Western blotting

Total cell protein was extracted with RIPA lysis buffer (Beyotime), and their concentrations were determined using a BCA protein detection kit (Thermo Fisher Scientific). Next, proteins were separated by PAGE (12%) and transferred to PVDF membranes. After blocking with skim milk (5%, w/v) for 2 h at 25°C, membranes were incubated with the following primary antibodies overnight at 4°C: SLC7A7 (1: 1000, Abcam, Cambridge, UK, ab236669), MYC (1: 1000, Cell Signaling Technology, MA, the USA, 18583), ACSS2 (1: 1000, Cell Signaling Technology, MA, the USA, 3658), and GAPDH (1: 1000, ABclonal, Wuhan, China, AC001). Membranes were then incubated with HRP-labeled IgG secondary antibody (1:2000, Beyotime, #Shanghai, China, A02080) for 2 h at 25°C. Protein bands on the membrane were then visualized using the ECL Plus kit (Meilunbio, Dalian, China). Finally, the band intensity was quantified *via* Image Lab 3.0 software (BioRad, Hercules, CA, USA).

### Statistical analysis

All statistical analysis was conducted using R software (Version: 3.6.1) and GraphPad Prism (Version: 7.00). We used a *t*-test to calculate the difference of continuous variables between binary groups. In contrast, the Pearson chi-square test was used to compare the categorical variables. The Mann–Whitney test was used when comparing the immune cell infiltration and immune pathway activation between the two groups. Western blot data were expressed as *M* ± *SE*, and analysis of variance (ANOVA) was used to compare differences between the two groups. *P* < 0.05 was considered statistically significant.

## Results

### Screening and functional analysis of fatty acid and lactate metabolism–related DEGs

**Figure 1:**
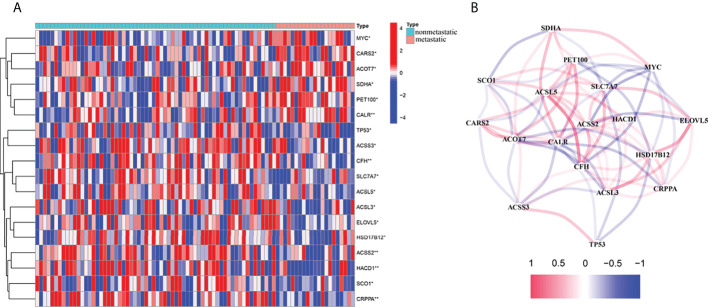
Expressions of the 18 fatty acid and lactate metabolism–related genes and the interactions among them. **(A)** Heat map (blue: low expression level; red: high expression level) of the fatty acid and lactate metabolism–related genes between the non-metastatic (nonmetastatic, brilliant blue) and the metastatic tissues (metastatic, red). *P*-values were showed as *P < 0.05; **P < 0.01. **(B)** The correlation network of these genes (Cutoff = 0.1; red line: positive correlation; blue line: negative correlation).

The 345 fatty acid metabolism– and lactate metabolism–related gene expression levels were compared in the pooled TARGET data from metastatic and non-metastatic tissues. We identified 18 DEGs. The RNA expression levels of these genes are shown in **Figure 1A**. We conducted a correlation analysis to investigate further the interactions between these fatty acid and lactate metabolism–related genes. We set the minimum required interaction score for the correlation analysis at 0.1, and we determined that DEGs (SDHA, SCO1, PET100, MYC, CARS2, ACSL5, SLC7A7, ACOT7, CALR, ACSS2, HACD1, ELOVL5, ACSS3, CFH, HSD17B12, ACSL3, CRPPA, and TP53) were hub genes (**Figure 1B**).

### Cancer classification based on the DEGs

**Figure 2:**
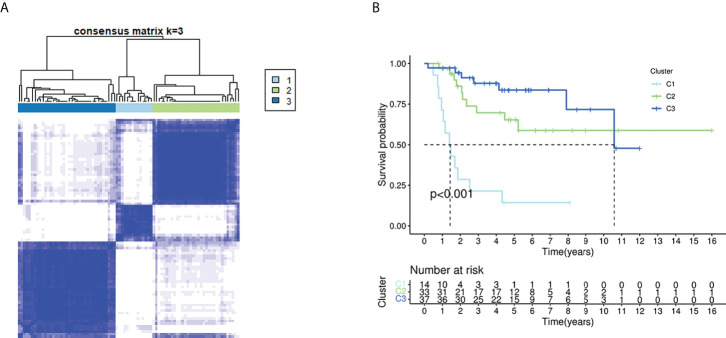
Tumor classification based on the fatty acid and lactate metabolism–related DEGs. **(A)** Eighty-four OS patients were grouped into three clusters according to the consensus clustering matrix (*k* = 3). **(B)** Kaplan–Meier OS curves for the three clusters.

When the clustering variable (*k*) was increased from 2 to 10, the intragroup correlations were decreased. So, when *k* = 3, the osteosarcoma patients could be divided into three clusters based on the 18 fatty acid metabolism– and lactate metabolism–related DEGs (**Figure 2A**). The survival rate was then compared between the three clusters, and we found a significant difference in survival rate between these clusters of patients (**Figure 2B**).

### Development of a prognostic fatty acid metabolism and lactate metabolism risk score

**Figure 3:**
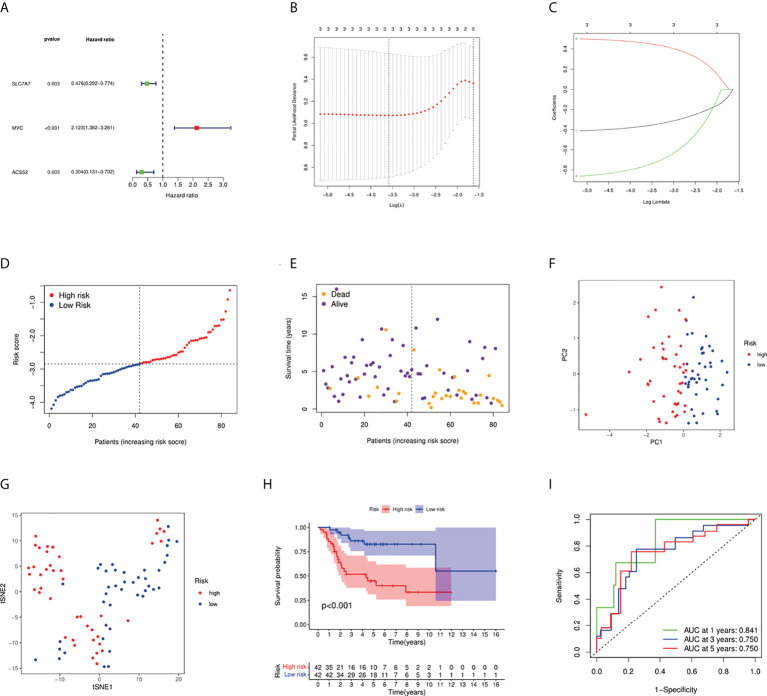
Construction of risk signature in the TARGET cohort. **(A)** Univariate Cox regression analysis of overall survival for each fatty acid and lactate metabolism–related gene, and three genes with *P* < 0.05. **(B)** Cross-validation for tuning the parameter selection in the LASSO regression. **(C)** LASSO regression of the three overall survival-related genes. **(D)** Distribution of patients based on the risk score. **(E)** The survival status of low-risk and high-risk population. **(F)** PCA plot for osteosarcoma patients based on the risk score. **(G)** The t-SNE analysis based on the risk score. **(H)** Kaplan–Meier curves for the overall survival of patients in the high- and low-risk groups. **(I)** ROC curves demonstrated the predictive efficiency of the risk score.

We firstly used univariate Cox regression to screen the genes related to survival. Three genes (SLC7A7, MYC, and ACSS2) met the *P* < 0.05. As shown in **Figure 3A**, MYC was a risk factor, whereas other genes were protective factors. Then, we conducted the LASSO regression analysis, and according to the optimum λ value, a 3-gene signature was constructed (**Figures 3B, C**). The risk score was calculated as follows: risk score = (-0.360* SLC7A7 exp.) + (0.454*MYC exp.) + (-0.749* ACSS2 exp.). As shown in **Figures 3D, E**, patients were divided into low- and high-risk subgroups based on the median risk score. The results of principal components analysis (PCA) and t-distributed stochastic neighbor embedding (t-SNE) indicated that patients with high or low risk were separated into two groups that had significantly different survival times (**Figures 3F, G**). Kaplan–Meier survival curve showed the prognosis was much worse in the high-risk group (**Figure 3H**, *P* < 0.001). The AUC values were higher than 0.7 for 1-, 3-, and 5-year survival predictions (**Figure 3I**).

### External validation of the fatty acid and lactate metabolism risk score

**Figure 4:**
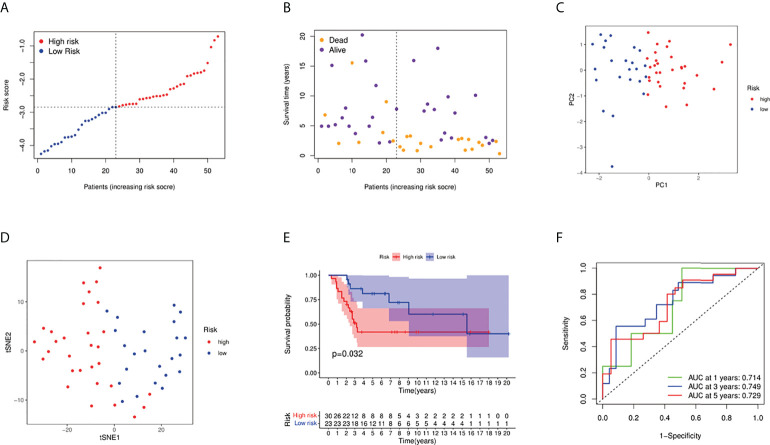
Validation of the risk model in the GEO cohort. **(A)** Distribution of patients in the GEO cohort based on the median risk score in the TARGET cohort. **(B)** The survival status of low-risk and high-risk population. **(C)** PCA plot for osteosarcoma patients. **(D)** The t-SNE analysis based on the risk score. **(E)** Kaplan–Meier curves for comparison of the overall survival between low- and high-risk groups; **(F)** Time-dependent ROC curves for osteosarcoma patients.

Fifty-three osteosarcoma patients from a GEO cohort (GSE21257) were extracted as the external validation cohort. Based on the above risk score, the validation cohort was divided into high- and low-risk groups (**Figures 4A, B**). As shown in **Figures 4C** and **4D**, the PCA and t-SNE results showed a satisfactory separation between the two groups. In addition, the Kaplan–Meier curve results also indicated a significant difference in prognosis between the two groups (**Figure 4E**, *P* = 0.032). AUC values of external validation also stated a good prediction performance for 1-, 3-, and 5-year survival (**Figure 4F**).

### The expression levels of three fatty acid and lactate metabolism–related genes in osteosarcoma

**Figure 5:**
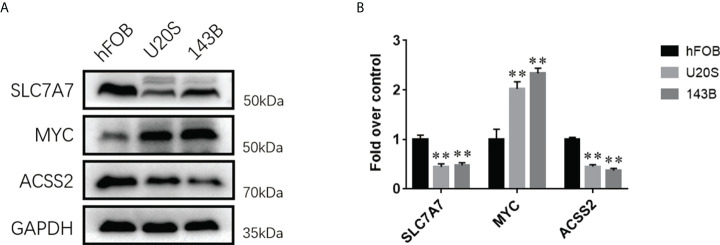
The expression levels of fatty acid and lactate metabolism related genes between osteosarcoma cell lines and osteoblasts. **(A)** Western blotting analysis of the expressions of SLC7A7, MYC, and ACSS2 proteins in hFOB, U20S, and 143B groups. GAPDH serves as an internal standard. The gels have been run under the same experimental conditions. **(B)** A histogram of the OD values of SLC7A7, MYC, and ACSS2 in each group (*n* = 3 per group). The obtained data are represented as *M* ± *SE*. Significance: **p < 0.01 versus hFOB group.

To demonstrate the importance and relevance of the genes in osteosarcoma, we further used Western blotting analysis to investigate the expression of three fatty acid and lactate metabolism–related genes between osteoblasts and osteosarcoma cells. Results showed that the expression levels of SLC7A7 and ACSS2 were significantly decreased in two osteosarcoma cell groups (U20S and 143B) compared with the osteoblast cell group (hFOB), whereas MYC was up-regulated in osteosarcoma groups (**Figures 5A, B**).

### Development of a nomogram based on fatty acid and lactate metabolism risk score

**Figure 6:**
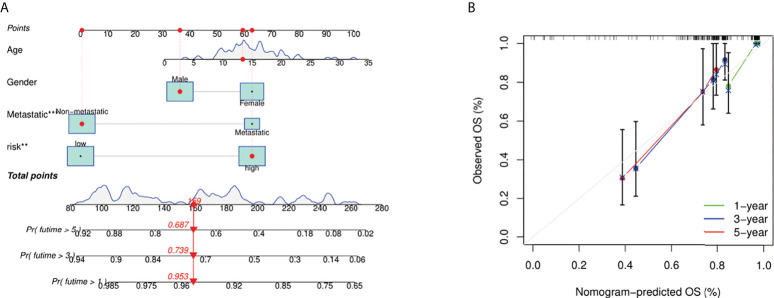
Construction and calibration of nomogram. **(A)** Nomogram integrating risk score and clinical characteristics. **(B)** Calibration of the nomogram at 1-, 3-, and 5-year survival in the TARGET cohort.

We integrated the fatty acid metabolism and lactate metabolism risk score with other clinical factors (age, gender, and metastatic status) to build a nomogram for prognosis prediction (**Figure 6A**). The curves indicated this prognosis prediction nomogram with excellent performance. 1-, 3-, and 5-year calibration curves showed that the nomogram had a superb consistency in predicting the survival rate (**Figure 6B**).

### Independent prognostic value of the risk model

**Figure 7:**
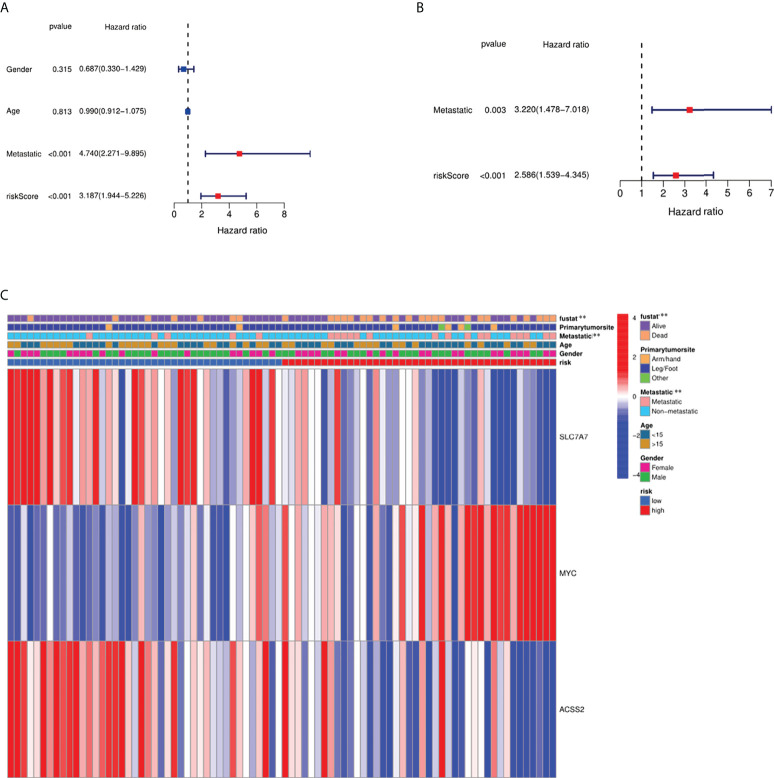
Independence detection of the constructed risk prediction model. **(A)** Univariate analysis for the TARGET cohort (gender: age, metastatic). **(B)** Multivariate analysis for the TARGET cohort. **(C)** Heat map (blue: low expression; red: high expression) for the connections between clinicopathologic features and the risk groups. **p < 0.01.

We conducted univariate and multivariable Cox regression analyses to evaluate the significance of risk score for prognostic prediction. The results of Cox regression analysis indicated that the risk score was significantly associated with prognosis (**Figures 7A, B**). A heat map was built to show the difference between fatty acid and lactate metabolism–related gene expression and clinical features in different groups (**Figure 7C**). The clinical features of osteosarcoma patients were shown in **Supplementary T1**.

### Functional analyses based on the risk model

**Figure 8:**
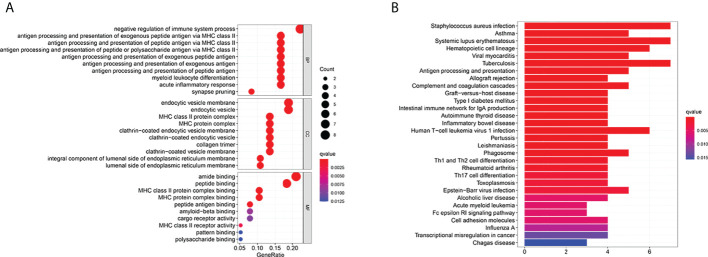
Functional analysis based on the DEGs between the two-risk groups in the TARGET cohort. **(A)** Bubble graph for GO enrichment (the bigger bubble means the more genes enriched, and the increasing depth of red means the differences were more obvious; *q*-value: the adjusted *p*-value). **(B)** Barplot graph for KEGG pathways (the longer bar means the more genes enriched, and the increasing depth of red means the differences were more obvious).

Thirty-eight DEGs between the low- and high-risk groups were identified. Among them, eight genes were up-regulated in the high-risk group, whereas 30 genes were down-regulated (**Supplementary T2**). We performed GO enrichment and KEGG pathway analysis based on these DEGs (**Figures 8A, B**). We found that immune regulation (negative regulation of immune system process, antigen process and presentation of antigen, endocytic vesicle and membrane, MHC class II–related pathways, and T-cell differentiation) were significantly enriched. The results showed that DEGs were mainly related to the immune system process, inflammatory response, and immune-related signaling pathways.

### Immune status and tumor microenvironment

**Figure 9:**
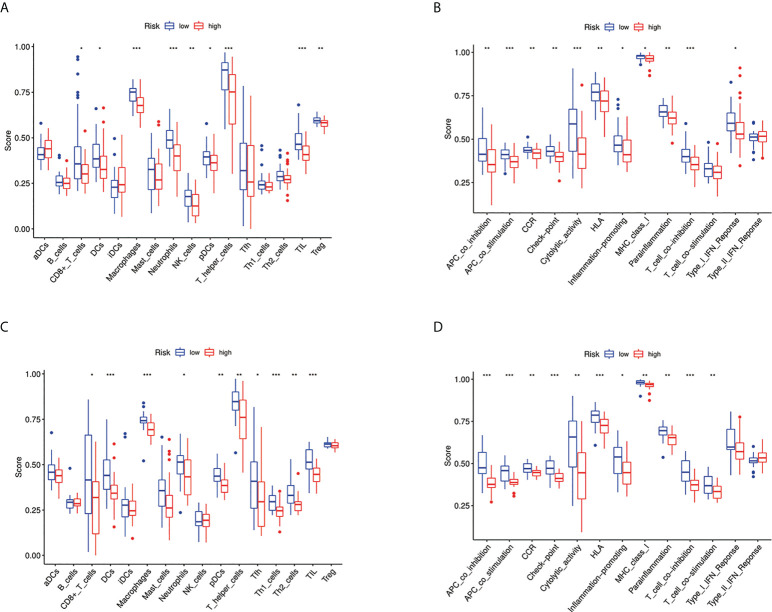
Immune status between different risk groups and the association between risk score and tumor microenvironment. **(A)** Comparison of the enrichment scores of 16 types of immune cells between low- (blue box) and high-risk (red box) group in the TARGET cohort. *p < 0.05, **p < 0.01, and ***p < 0.001; **(B)** Comparison of the enrichment scores of 13 types of immune functions between low- (blue box) and high-risk (red box) group in the TARGET cohort. *p < 0.05, **p < 0.01, and ***p < 0.001; **(C)** Comparison of the enrichment scores of 16 types of immune cells between low- (blue box) and high-risk (red box) group in the GEO cohort. *p < 0.05, **p < 0.01, and ***p < 0.001; **(D)** Comparison of the enrichment scores of 13 types of immune functions between low- (blue box) and high-risk (red box) group in the GEO cohort. *p < 0.05, **p < 0.01, and ***p < 0.001.

**Figure 10:**
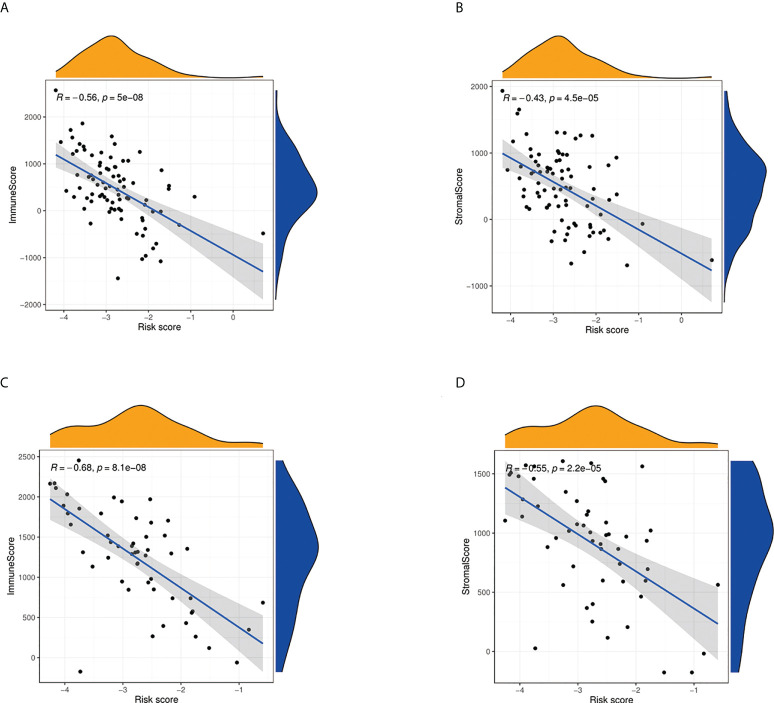
Estimate analysis for osteosarcoma patients. **(A)** The relationship between risk score and immune score in the TARGET cohort. **(B)** The relationship between risk score and stromal score in TARGET cohort. **(C)** The relationship between risk score and immune score in the GEO cohort. **(D)** The relationship between risk score and stromal score in the GEO cohort.

We further used ssGSEA to evaluate the enrichment scores of 16 types of immune cells and the activity of 13 immune-related pathways between the high- and low-risk groups in two cohorts. Results showed that CD8^+^ T cells, dendritic cells (DCS), macrophages, neutrophils, natural killer (NK) cells, pDCs, T helper cells, tumor-infiltrating lymphocytes (TIL), and T_reg_ were significantly different between the low-risk and high-risk groups. The scores of immune cells were lower in the high-risk group (**Figure 9A**). Moreover, we indicated that the immune scores of antigen presenting cell (APC) co-inhibition, antigen presenting cell (APC) co-stimulation, CCR, checkpoint, cytolytic activity, HLA, inflammation-promoting, MHC, para inflammation, T-cell co-inhibition, and type I IFN response were significantly higher in high-risk group (**Figure 9B**). The enrichment scores of these immune cells and the immune pathways were also lower in the high-risk group in the GEO cohort (**Figures 9C, D**). Our results may explain the more significant the threat of osteosarcoma to the body, the easier it is to weaken the immune response. The Spearman correlation analysis was used to evaluate further the relationship between stromal, immune, and risk scores. We found that the risk score has a negative correlation with the immune score in the TARGET cohort (*p* < 0.05; **Figure 10A**), and the risk score was also negatively associated with the stromal score (*p* < 0.05; **Figure 10B**). In addition, we draw similar conclusions in the GEO cohort (**Figures 10C, D**).

### Drug susceptibility analysis

**Figure 11:**
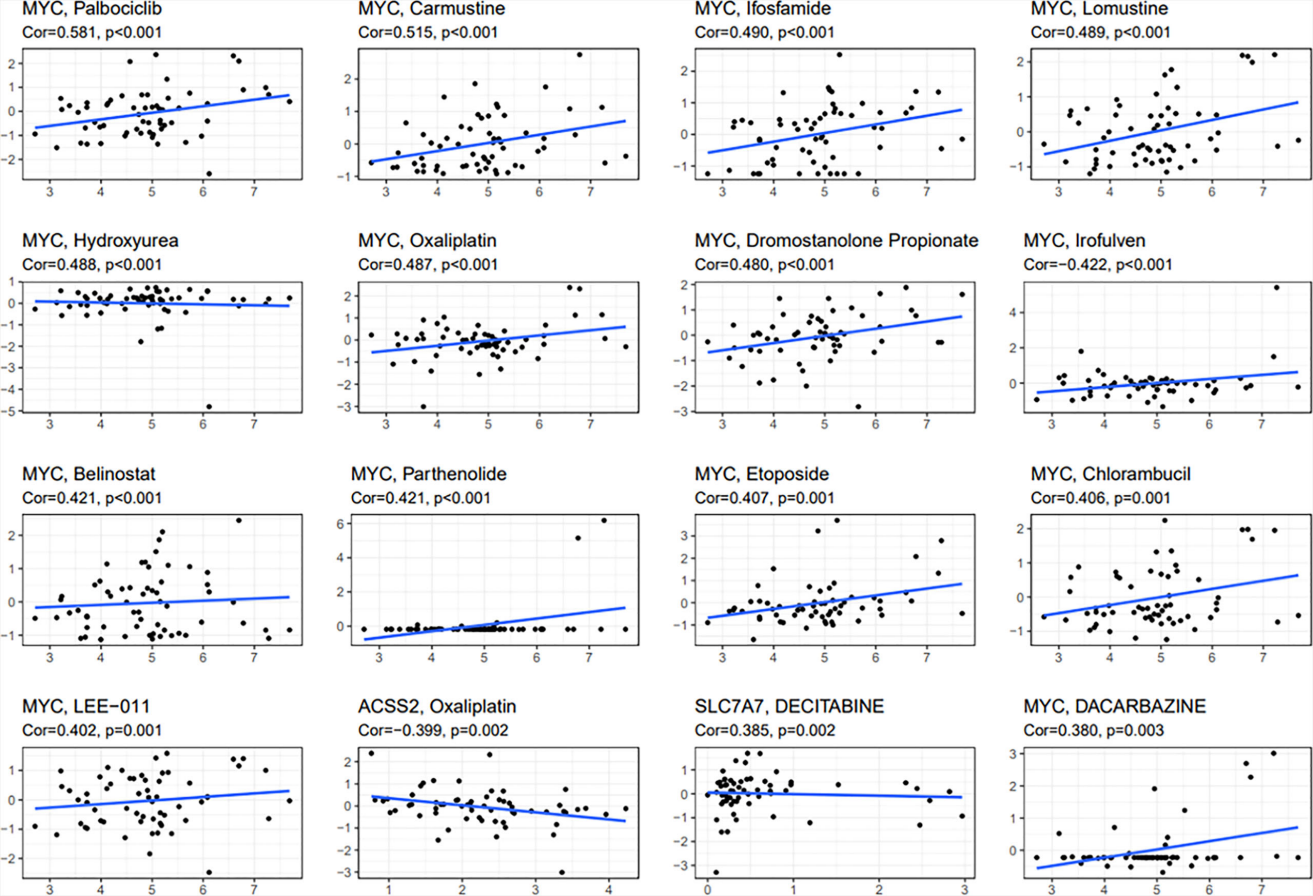
Scatter plot of relationship between prognostic gene expression and drug sensitivity. The top 16 correlation analyses are shown based on the *p*-value.

To study the sensitivity of prognostic genes to chemotherapeutic drugs, we downloaded data from the NCI-60 panel of human cancer cell lines. We investigated the association between three fatty acid and lactate metabolism–related genes and common anticancer drug sensitivity (**Supplementary T3**). The top 16 correlation analysis results are provided based on the *p*-value (**Figure 11**). MYC is an important therapeutic target, which is sensitive to irofulven, dromostanolone propionate, oxaliplatin, hydroxyurea, belinostat, parthenolide, etoposide, chlorambucil, lomustine, ifosfamide, carmustine, palbociclib, dacarbazine, and LEE-011 (all *p* < 0.005). The expression of ACSS2 is insensitive to oxaliplatin (*p* = 0.002). Moreover, SLC7A7 is sensitive to decitabine (*p* = 0.002).

## Discussion

Osteosarcoma is the most frequent solid malignancy of bone and with high metastatic potential. The prognosis of osteosarcoma patients with metastasis is inferior. Many risk score systems for prognostic prediction have been developed for cancer patients. Recently, fatty acid metabolism and lactate metabolism in cancer cells have received increasing attention. Fatty acid metabolism regulation can meet energy demands and affect cancer cell proliferation, growth, and transformation (18). Lactic acid is critical for epigenetic modification and DNA repair in cancer cells (19). Some scholars have shown that reducing the production and output of lactic acid in the extracellular environment can weaken the driving or maintenance of chemoresistant characteristics of tumor cells (20). Low extracellular pH has many benefits for the survival of tumor cells, including chemotherapy resistance (21). The above two metabolisms were significantly associated with cancer progression. Therefore, it is imperative to comprehensively investigate fatty acid and lactate metabolism to predict the outcomes and therapeutic responses for osteosarcoma patients with metastasis. Prognostic prediction is critical in clinical applications and improves patients’ prognostic management. As far as we know, this is the first study developing a risk score model for predicting prognosis and therapeutic efficacy for osteosarcoma patients.

We identified 18 DEGs; three were determined to construct a risk score model using LASSO and Cox regression analyses. The performance of this model was confirmed by internal and external validation, which showed a robust survival prediction performance. In addition, a nomogram was constructed *via* the integration of the fatty acid metabolism and lactate metabolism–based risk scores with clinical factors (age, gender, and metastatic status), which could help predict the survival of patients and guide the follow-up of individual treatments.

Our present study constructed a novel fatty acid and lactate metabolism–risk score model including three genes (SLC7A7, MYC, and ACSS2). SLC7A7 (solute carrier family 7, amino acid transporter light chain, y + L system, member 7) is a critical gene in the regulation of cationic amino acid transport (22). Mutations in SLC7A7 may cause transporter dysfunction (23). Overexpression of SLC7A7 is correlated with poor prognosis in patients with glioblastoma (24). Besides, SLC7A7 has been highly expressed in chemotherapy-resistant ovarian cancer and is associated with chemotherapy outcomes (25). More importantly, the expression of SLC7A7 was significantly increased in monocytes during macrophage differentiation (26). However, the role of SLC7A7 in osteosarcoma progression and immunology is still unclear (27). Our study pointed out that the expression of SLC7A7 is significantly associated with the prognosis of osteosarcoma. Our study showed that the expression level of SLC7A7 was significantly decreased in osteosarcoma groups based on Western blot results. We speculated that the proliferation and migration of osteosarcoma cells can down-regulate their SLC7A7 expression. MYC is a regulator of gene transcription and controls a diverse set of biological programs (28, 29). MYC can promote programs of proliferative cell growth; thus, MYC is frequently up-regulated in tumors (30). MYC is associated with many cancers’ progression (31). For example, a previous study showed that MYC was important in lung tumor progression, maintenance, and therapeutic resistance (32). Western blotting analysis revealed that the level of MYC was up-regulated in osteosarcoma. Therefore, targeting MYC to regulate transcriptional programs may be an attractive therapeutic intervention. Acetyl-CoA is a crucial metabolite for many cellular processes, including fatty acid synthesis, ATP production, and protein acetylation (33). Acetyl-CoA synthetase 2 (ACSS2) is an enzyme that converts acetate to acetyl-CoA (34). ACSS2 regulates cell cycle progression and metabolic reprogramming of tumor cells by stimulating the acetylation of histones and transcription factors (35). A recent study has pointed out that cancer cells up-regulate ACSS2, which may cause by responding to stresses such as low nutrient availability and hypoxia (33). However, some studies have indicated that the decrease of ACSS2 can promote tumor progression, and promoting the expression of ACSS2 can inhibit tumor growth and development (36, 37). ACSS2 was rarely researched in osteosarcoma. Our study showed that ACSS2 might play an essential role in the prognosis of osteosarcoma. Our findings revealed that the expression of ACSS2 was down-regulated in osteosarcoma cells. ACSS2 could be a new potential biomarker for early diagnosis and subsequent treatment of osteosarcoma. We believe these three genes may play an important role in osteosarcoma’s occurrence, development, and prognosis.

According to GO and KEGG analysis results, we can reasonably infer that fatty acid and lactate metabolism–related genes are related to the tumor immune microenvironment. Infiltrating immune cells are significant for tumor growth, invasion, and metastasis. Therefore, it may be a promising therapeutic target (38). The low level of critical anti-tumor infiltrating immune cells indicates an overall impairment of immune functions in high-risk patients in the TARGET cohort. The same conclusion was also verified in the GEO cohort. Compared with the low-risk group, the activation of significant immune pathways decreased in the high-risk group. The risk score was significantly correlated with the immune score and stromal score, which means that immunity and tumor environment may inhibit the aggression of osteosarcoma. Based on these findings, the weakening of anti-tumor immunity and immune environment leads to the poor survival outcome of high-risk osteosarcoma patients. Because osteosarcoma patients’ immune cells and immune environment are damaged, the body cannot identify and kill tumor cells. Finally, it forms a substantial sarcoma that can be detected by imaging.

According to the data analysis of 60 different cell lines, the increased expression of these predictive genes enhanced drug sensitivity or the resistance to chemotherapy drugs approved by the Food and Drug Administration. For example, cancer cells were sensitive to oxaliplatin with the elevated expression of MYC, whereas they were insensitive with the increased expression of ACSS2. MYC upregulation is a valuable biomarker for neoadjuvant chemotherapy in primary colorectal cancer with liver metastasis (39). These findings might be excellent markers for assessing the response to targeted therapy to facilitate the development of personalized treatment for osteosarcoma.

However, in our present study, some limitations should be noted. First, our results were constructed and validated retrospectively according to data from public databases. Therefore, future research is needed to evaluate the clinical utility of our risk score model in patients with osteosarcoma. Moreover, comprehensive functional experiments are also required to show the elusive mechanisms of the three fatty acid metabolism and lactate metabolism–related genes, which will be conducted in our future research.

## Conclusions

Our study was the first to identify the differentially expressed fatty acid and lactate metabolism–related genes in osteosarcoma. According to the differential expression genes, we built a risk score model and nomogram for osteosarcoma patients to predict the prognosis, which has a critical role in clinical applications and improves patients’ prognostic management. We also found the adjustment of immune cells and immune environment in high-risk groups, which could provide potential immunotherapy for further research. Besides, cancer cells with predictive genes are sensitive or insensitive to chemotherapeutics, which may offer a new sight for targeted treatment of osteosarcoma in the future.

## Data availability statement

The original contributions presented in the study are included in the article/**Supplementary Material**. Further inquiries can be directed to the corresponding authors.

## Author contributions

XW and JX conceived the ideas. All authors designed, performed, and analyzed the experiments. ZW, TH, and HS carried out the data analysis and wrote the manuscript. All authors reviewed and approved the final manuscript.

## Funding

Funding from Zhuji Affiliated Hospital of Wenzhou Medical University.

## Acknowledgments

We like to acknowledge the TARGET and the GEO (GSE21257) network for providing data. At the same time, we thank Zhuji Affiliated Hospital of Wenzhou Medical University for its financial support.

## Conflict of interest

The authors declare that the research was conducted in the absence of any commercial or financial relationships that could be construed as a potential conflict of interest.

## Publisher’s note

All claims expressed in this article are solely those of the authors and do not necessarily represent those of their affiliated organizations, or those of the publisher, the editors and the reviewers. Any product that may be evaluated in this article, or claim that may be made by its manufacturer, is not guaranteed or endorsed by the publisher.

## References

[1] Mirabello L Troisi RJ Savage SA. Osteosarcoma incidence and survival rates from 1973 to 2004: data from the surveillance, epidemiology, and end results program. Cancer. (2009) 115(7):1531–43. doi: 10.1002/cncr.24121 PMC2813207 19197972

[2] Ritter J Bielack SS. Osteosarcoma. Ann Oncol (2010) 21 Suppl 7:vii320–5. doi: 10.1093/annonc/mdq276 20943636

[3] Gill J Gorlick R. Advancing therapy for osteosarcoma. Nat Rev Clin Oncol (2021) 18(10):609–24. doi: 10.1038/s41571-021-00519-8 34131316

[4] Kager L Zoubek A Potschger U Kastner U Flege S Kempf-Bielack B. Primary metastatic osteosarcoma: presentation and outcome of patients treated on neoadjuvant cooperative osteosarcoma study group protocols. J Clin Oncol (2003) 21(10):2011–8. doi: 10.1200/JCO.2003.08.132 12743156

[5] Eaton BR Schwarz R Vatner R Yeh B Claude L Indelicato DJ. Osteosarcoma. Pediatr Blood Cancer (2021) 68 Suppl 2:e28352. doi: 10.1002/pbc.28352 32779875

[6] Yu T Wang Y Fan Y Fang N Wang T Xu T. CircRNAs in cancer metabolism: a review. J Hematol Oncol (2019) 12(1):90. doi: 10.1186/s13045-019-0776-8 31484561 PMC6727394

[7] Yi M Li J Chen S Cai J Ban Y Peng Q. Emerging role of lipid metabolism alterations in cancer stem cells. J Exp Clin Cancer Res (2018) 37(1):118. doi: 10.1186/s13046-018-0784-5 29907133 PMC6003041

[8] Carracedo A Cantley LC Pandolfi PP. Cancer metabolism: fatty acid oxidation in the limelight. Nat Rev Cancer (2013) 13(4):227–32. doi: 10.1038/nrc3483 PMC3766957 23446547

[9] Currie E Schulze A Zechner R Walther TC Farese RV Jr. Cellular fatty acid metabolism and cancer. Cell Metab (2013) 18(2):153–61. doi: 10.1016/j.cmet.2013.05.017 PMC3742569 23791484

[10] Doherty JR Cleveland JL. Targeting lactate metabolism for cancer therapeutics. J Clin Invest (2013) 123(9):3685–92. doi: 10.1172/JCI69741 PMC3754272 23999443

[11] Certo M Tsai CH Pucino V Ho PC Mauro C. Lactate modulation of immune responses in inflammatory versus tumour microenvironments. Nat Rev Immunol (2021) 21(3):151–61. doi: 10.1038/s41577-020-0406-2 32839570

[12] Izzo LT Wellen KE. Histone lactylation links metabolism and gene regulation. Nature. (2019) 574(7779):492–3. doi: 10.1038/d41586-019-03122-1 31645737

[13] Yang C Huang X Liu Z Qin W Wang C. Metabolism-associated molecular classification of hepatocellular carcinoma. Mol Oncol (2020) 14(4):896–913. doi: 10.1002/1878-0261.12639 31955511 PMC7138397

[14] Liberzon A Birger C Thorvaldsdottir H Ghandi M Mesirov JP Tamayo P. The molecular signatures database (MSigDB) hallmark gene set collection. Cell Syst (2015) 1(6):417–25. doi: 10.1016/j.cels.2015.12.004 PMC4707969 26771021

[15] Sun Z Tao W Guo X Jing C Zhang M Wang Z. Construction of a lactate-related prognostic signature for predicting prognosis, tumor microenvironment, and immune response in kidney renal clear cell carcinoma. Front Immunol (2022) 13:818984. doi: 10.3389/fimmu.2022.818984 35250999 PMC8892380

[16] Ye Y Dai Q Qi H. A novel defined pyroptosis-related gene signature for predicting the prognosis of ovarian cancer. Cell Death Discovery (2021) 7(1):71. doi: 10.1038/s41420-021-00451-x 33828074 PMC8026591

[17] Yoshihara K Shahmoradgoli M Martinez E Vegesna R Kim H Torres-Garcia W. Inferring tumour purity and stromal and immune cell admixture from expression data. Nat Commun (2013) 4:2612. doi: 10.1038/ncomms3612 24113773 PMC3826632

[18] Jin Z Chai YD Hu S. Fatty acid metabolism and cancer. Adv Exp Med Biol (2021) 1280:231–41. doi: 10.1007/978-3-030-51652-9_16 33791986

[19] Wagner W Kania KD Ciszewski WM. Stimulation of lactate receptor (HCAR1) affects cellular DNA repair capacity. DNA Repair (Amst) (2017) 52:49–58. doi: 10.1016/j.dnarep.2017.02.007 28258841

[20] Soni VK Shukla D Kumar A Vishvakarma NK. Curcumin circumvent lactate-induced chemoresistance in hepatic cancer cells through modulation of hydroxycarboxylic acid receptor-1. Int J Biochem Cell Biol (2020) 123:105752. doi: 10.1016/j.biocel.2020.105752 32325281

[21] Estrella V Chen T Lloyd M Wojtkowiak J Cornnell HH Ibrahim-Hashim A. Acidity generated by the tumor microenvironment drives local invasion. Cancer Res (2013) 73(5):1524–35. doi: 10.1158/0008-5472.CAN-12-2796 PMC3594450 23288510

[22] He L Vasiliou K Nebert DW. Analysis and update of the human solute carrier (SLC) gene superfamily. Hum Genomics (2009) 3(2):195–206. doi: 10.1186/1479-7364-3-2-195 19164095 PMC2752037

[23] Sperandeo MP Andria G Sebastio G. Lysinuric protein intolerance: update and extended mutation analysis of the SLC7A7 gene. Hum Mutat (2008) 29(1):14–21. doi: 10.1002/humu.20589 17764084

[24] Fan S Zhao Y Li X Du Y Wang J Song X. Genetic variants in SLC7A7 are associated with risk of glioma in a Chinese population. Exp Biol Med (2013) 238(9):1075–81. doi: 10.1177/1535370213498977 23975734

[25] Cheng L Lu W Kulkarni B Pejovic T Yan X Chiang J-H. Analysis of chemotherapy response programs in ovarian cancers by the next-generation sequencing technologies. Gynecol Oncol (2010) 117(2):159–69. doi: 10.1016/j.ygyno.2010.01.041 PMC2849907 20181382

[26] Barilli A Rotoli BM Visigalli R Bussolati O Gazzola GC Dall'Asta V. Arginine transport in human monocytic leukemia THP-1 cells during macrophage differentiation. J leukoc Biol (2011) 90(2):293–303. doi: 10.1189/jlb.0910510 21586674

[27] Dai W Feng J Hu X Chen Y Gu Q Gong W. SLC7A7 is a prognostic biomarker correlated with immune infiltrates in non-small cell lung cancer. Cancer Cell Int (2021) 21(1):106. doi: 10.1186/s12935-021-01781-7 33632211 PMC7905560

[28] Lin CY Lovén J Rahl PB Paranal RM Burge CB Bradner JE. Transcriptional amplification in tumor cells with elevated c-myc. Cell. (2012) 151(1):56–67. doi: 10.1016/j.cell.2012.08.026 23021215 PMC3462372

[29] Nie Z Hu G Wei G Cui K Yamane A Resch W. C-myc is a universal amplifier of expressed genes in lymphocytes and embryonic stem cells. Cell. (2012) 151(1):68–79. doi: 10.1016/j.cell.2012.08.033 23021216 PMC3471363

[30] Zaytseva O Kim NH Quinn LM. MYC in brain development and cancer. Int J Mol Sci (2020) 21(20). doi: 10.3390/ijms21207742 PMC7588885 33092025

[31] Lourenco C Resetca D Redel C Lin P MacDonald AS Ciaccio R. MYC protein interactors in gene transcription and cancer. Nat Rev Cancer (2021) 21(9):579–91. doi: 10.1038/s41568-021-00367-9 34188192

[32] Massó-Vallés D Beaulieu ME Soucek L. MYC, MYCL, and MYCN as therapeutic targets in lung cancer. Expert Opin Ther targets (2020) 24(2):101–14. doi: 10.1080/14728222.2020.1723548 32003251

[33] Miller KD Pniewski K Perry CE Papp SB Shaffer JD Velasco-Silva JN. Targeting ACSS2 with a transition-state mimetic inhibits triple-negative breast cancer growth. Cancer Res (2021) 81(5):1252–64. doi: 10.1158/0008-5472.can-20-1847 PMC8026699 33414169

[34] Schug ZT Peck B Jones DT Zhang Q Grosskurth S Alam IS. Acetyl-CoA synthetase 2 promotes acetate utilization and maintains cancer cell growth under metabolic stress. Cancer Cell (2015) 27(1):57–71. doi: 10.1016/j.ccell.2014.12.002 25584894 PMC4297291

[35] Ling R Chen G Tang X Liu N Zhou Y Chen D. Acetyl-CoA synthetase 2(ACSS2): a review with a focus on metabolism and tumor development. Discovery Oncol (2022) 13(1):58. doi: 10.1007/s12672-022-00521-1 PMC9263018 35798917

[36] Liang Y Yi L Deng P Wang L Yue Y Wang H. Rapamycin antagonizes cadmium-induced breast cancer cell proliferation and metastasis through directly modulating ACSS2. Ecotoxicol Environ Saf (2021) 224:112626. doi: 10.1016/j.ecoenv.2021.112626 34411822

[37] Hur H Kim YB Ham IH Lee D. Loss of ACSS2 expression predicts poor prognosis in patients with gastric cancer. J Surg Oncol (2015) 112(6):585–91. doi: 10.1002/jso.24043 26381042

[38] Pitt JM Marabelle A Eggermont A Soria JC Kroemer G Zitvogel L. Targeting the tumor microenvironment: removing obstruction to anticancer immune responses and immunotherapy. Ann Oncol (2016) 27(8):1482–92. doi: 10.1093/annonc/mdw168 27069014

[39] Kato T Matsuhashi N Tomita H Takahashi T Iwata Y Fukada M. MYC up-regulation is a useful biomarker for preoperative neoadjuvant chemotherapy combined with anti-EGFR in liver metastasis from colorectal cancer. In Vivo (2021) 35(1):203–13. doi: 10.21873/invivo.12249 PMC7880764 33402467

