# Supplementary material for: Comprehensive analysis of fatty acid and lactate metabolism–related genes for prognosis value, immune infiltration, and therapy in osteosarcoma patients
Source: Front Oncol. 2022 Sep 2;12:934080. doi: 10.3389/fonc.2022.934080 (PMC9478861; doi:10.3389/fonc.2022.934080)
Supplement: Supplementary file 1 [file DataSheet_1.zip › T1.docx]

| Variables (clinical features) | Options | High-risk patients(n) | Low-risk patients(n) |
| --- | --- | --- | --- |
| Gender | Male | 22 | 25 |
|  | Female | 20 | 17 |
| Age | <15 | 26 | 20 |
|  | ≥15 | 16 | 22 |
| Metastatic | No | 26 | 37 |
|  | Yes | 16 | 5 |
| Survival | Alive | 20 | 35 |
|  | Dead | 22 | 7 |
| Primary tumor site | Leg/foot | 36 | 40 |
|  | Arm/hand | 4 | 2 |
|  | Others | 2 | 0 |

T1 clinical features of osteosarcoma patients.
