# Supplementary figures and images for: Comprehensive analysis of fatty acid and lactate metabolism–related genes for prognosis value, immune infiltration, and therapy in osteosarcoma patients
Source: Front Oncol. 2022 Sep 2;12:934080. doi: 10.3389/fonc.2022.934080 (PMC9478861; doi:10.3389/fonc.2022.934080)

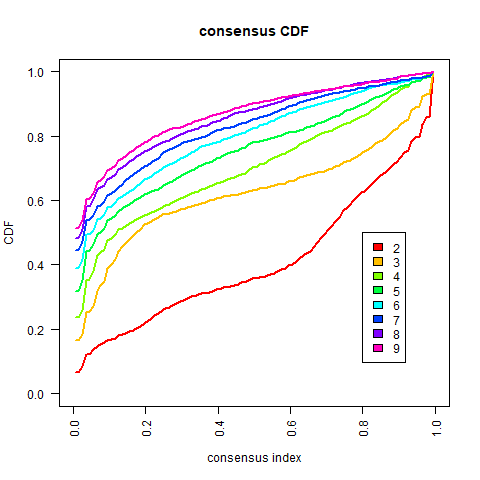

Supplement: Supplementary file 5 [file Image_1.png]
